# Editorial: 15 years of frontiers in neuroanatomy: the circuits behind the visual cortex

**DOI:** 10.3389/fnana.2024.1507122

**Published:** 2024-10-23

**Authors:** Toru Takahata, Song-Lin Ding

**Affiliations:** ^1^Systems Neuroscience Section, Center for the Evolutionary Origins of Human Behavior, Kyoto University, Kyoto, Japan; ^2^Allen Institute for Brain Science, Seattle, WA, United States

**Keywords:** visual cortex, superior colliculus, fovea, corpus callosum, vision-guided movement

In the special Research Topic “*15 Years of Frontiers in Neuroanatomy: The Circuits Behind the Visual Cortex*,” four original research articles have been published. Each study presents novel neuroanatomical concepts related to the visual system, spanning from the single-cell to meso-scale, systems-level insights. These studies utilize cutting-edge imaging and tracing techniques, offering readers valuable perspectives on recent technical advancements as well. While there are some scientific overlaps between the studies, they also complement and enhance each other's findings.

Hovde et al. studied how the brain in mice connects visual information to movement. The brain has two main pathways for processing visual information: one that helps with recognizing objects and another that helps guide movement. In mice, it's not completely clear how visual signals from the brain's primary visual area (V1) are sent to motor regions that control movement. To investigate this, the researchers used a technique to label and trace connections between V1 and motor-related areas of the brain. By examining and reconstructing these brain regions in 3D, they discovered that the strongest connections happened in specific regions surrounding the visual cortex. These connections were mostly found in a particular layer of the brain (layer 2/3). This study provides evidence that visual information in mice is passed to the motor cortex through these key regions, helping guide movement based on what the mouse sees.

Zhang et al. explored how fibers in the brain's corpus callosum are organized. While past studies have given mixed results about where these fibers connect in the brain, there hasn't been a full-brain analysis of certain callosal fiber bundles, called heterotopic callosal bundles (HeCBs), which link different regions between the hemispheres. Using advanced brain imaging data and analysis techniques from the Human Connectome Project, they mapped out these connections. The study showed that the fibers followed a specific pattern: they were arranged from front to back in segments that overlapped slightly due to the presence of HeCBs. These connections matched the natural layout of the brain before it developed its folded structure. Interestingly, the HeCBs were much stronger than the connections linking identical areas in each hemisphere. This research helps to better understand how the two sides of the brain communicate and could be important for preventing certain brain disorders where this communication is disrupted.

Jiang et al. studied how the primary visual cortex connects to different types of cells in the superior colliculus. The superior colliculus gets visual input both directly from the eyes and from the visual cortex, but it wasn't clear which specific cell types in this area receive input from the visual cortex. To find out, they identified four types of cells in the superficial layers of the superior colliculus based on their shape and electrical properties: wide-field, narrow-field, horizontal, and stellate cells. They used a technique that involved activating visual cortex fibers with light and recording the response in the superior colliculus cells. They found that all four types of cells received direct input from the visual cortex, but wide-field cells were more likely to receive this input. This study helps to clarify how the brain processes visual information by showing which specific cells in the superior colliculus are connected to the primary visual cortex.

Li et al. explored how different areas of the brain in macaques process visual information from the fovea. Although the fovea covers a small part of the visual field, a large portion of the brain's visual cortex is dedicated to processing this area because it's crucial for recognizing objects, controlling movement, and focusing attention. However, many earlier studies focused on areas surrounding the fovea (parafovea), which may have led to misunderstandings about how the brain structures work. In this study, researchers aimed to better understand how the brain areas responsible for the fovea are organized and connected. They injected specific tracers into two key visual areas (V1 and V4) of the brain and tracked how these areas communicate with each other. They found strong connections between V1 and V4 in the foveal region, whereas these connections were weaker in the parafoveal regions. The study also revealed that the foveal region has continuous connectivity across several visual areas, something that previous imaging studies missed. They observed that certain brain layers were responsible for sending and receiving signals between different areas, showing a complex network of communication within the foveal cortex. This research sheds new light on how the brain processes detailed visual information from the fovea, addressing gaps left by earlier studies.

These findings not only deepen our knowledge of how visual information is processed and integrated across different regions of the brain, but also offer new insights into how these systems might be disrupted in disease. As research continues to build upon these foundational studies, we anticipate that the exploration of visual circuits will further expand, leading to new discoveries that can address both basic neuroscience questions and clinical challenges. We hope that readers find this special collection both informative and inspiring, and that these contributions advance the field of neuroanatomy and enhance future research in visual neuroscience.

